# Emergence of SARS-CoV-2 B.1.1.7 Lineage — United States, December 29, 2020–January 12, 2021

**DOI:** 10.15585/mmwr.mm7003e2

**Published:** 2021-01-22

**Authors:** Summer E. Galloway, Prabasaj Paul, Duncan R. MacCannell, Michael A. Johansson, John T. Brooks, Adam MacNeil, Rachel B. Slayton, Suxiang Tong, Benjamin J. Silk, Gregory L. Armstrong, Matthew Biggerstaff, Vivien G. Dugan

**Affiliations:** ^1^CDC COVID-19 Response Team; ^2^Office of Advanced Molecular Detection, National Center for Emerging and Zoonotic Infectious Diseases, CDC.

On December 14, 2020, the United Kingdom reported a SARS-CoV-2 variant of concern (VOC), lineage B.1.1.7, also referred to as VOC 202012/01 or 20I/501Y.V1.* The B.1.1.7 variant is estimated to have emerged in September 2020 and has quickly become the dominant circulating SARS-CoV-2 variant in England (*1*). B.1.1.7 has been detected in over 30 countries, including the United States. As of January 13, 2021, approximately 76 cases of B.1.1.7 have been detected in 12 U.S. states.^†^ Multiple lines of evidence indicate that B.1.1.7 is more efficiently transmitted than are other SARS-CoV-2 variants (*1*–*3*). The modeled trajectory of this variant in the U.S. exhibits rapid growth in early 2021, becoming the predominant variant in March. Increased SARS-CoV-2 transmission might threaten strained health care resources, require extended and more rigorous implementation of public health strategies (*4*), and increase the percentage of population immunity required for pandemic control. Taking measures to reduce transmission now can lessen the potential impact of B.1.1.7 and allow critical time to increase vaccination coverage. Collectively, enhanced genomic surveillance combined with continued compliance with effective public health measures, including vaccination, physical distancing, use of masks, hand hygiene, and isolation and quarantine, will be essential to limiting the spread of SARS-CoV-2, the virus that causes coronavirus disease 2019 (COVID-19). Strategic testing of persons without symptoms but at higher risk of infection, such as those exposed to SARS-CoV-2 or who have frequent unavoidable contact with the public, provides another opportunity to limit ongoing spread.

Global genomic surveillance and rapid open-source sharing of viral genome sequences have facilitated near real-time detection, comparison, and tracking of evolving SARS-CoV-2 variants that can inform public health efforts to control the pandemic. Whereas some mutations in the viral genome emerge and then recede, others might confer a selective advantage to the variant, including enhanced transmissibility, so that such a variant can rapidly dominate other circulating variants. Early in the pandemic, variants of SARS-CoV-2 containing the D614G mutation in the spike (S) protein that increases receptor binding avidity rapidly became dominant in many geographic regions (*5*,*6*).

In late fall 2020, multiple countries reported detecting SARS-CoV-2 variants that spread more efficiently. In addition to the B.1.1.7 variant, notable variants include the B.1.351 lineage first detected in South Africa and the recently identified B.1.1.28 subclade (renamed “P.1”) detected in four travelers from Brazil during routine screening at the Haneda (Tokyo) airport.^§^ These variants carry a constellation of genetic mutations, including in the S protein receptor-binding domain, which is essential for binding to the host cell angiotensin-converting enzyme-2 (ACE-2) receptor to facilitate virus entry. Evidence suggests that other mutations found in these variants might confer not only increased transmissibility but might also affect the performance of some diagnostic real-time reverse transcription–polymerase chain reaction (RT-PCR) assays^¶^ and reduce susceptibility to neutralizing antibodies (*2*,*3*,*5*–*10*). A recent case report documented the first case of SARS-CoV-2 reinfection in Brazil with a SARS-CoV-2 variant that contained the E484K mutation,** which has been shown to reduce neutralization by convalescent sera and monoclonal antibodies (*9*,*10*).

This report focuses on the emergence of the B.1.1.7 variant in the United States. As of January 12, 2021, neither the B.1.351 nor the P.1 variants have been detected in the United States. For information about emerging SARS-CoV-2 variants of concern, CDC maintains a webpage dedicated to providing information on emerging SARS-CoV-2 variants.^††^

## B.1.1.7 lineage (20I/501Y.V1)

The B.1.1.7 variant carries a mutation in the S protein (N501Y) that affects the conformation of receptor-binding domain. This variant has 13 other B.1.1.7 lineage-defining mutations (Table), several of which are in the S protein, including a deletion at positions 69 and 70 (del69–70) that evolved spontaneously in other SARS-CoV-2 variants and is hypothesized to increase transmissibility (*2*,*7*). The deletion at positions 69 and 70 causes S-gene target failure (SGTF) in at least one RT-PCR–based diagnostic assay (i.e., with the ThermoFisher TaqPath COVID-19 assay, the B.1.1.7 variant and other variants with the del69–70 produce a negative result for S-gene target and a positive result for the other two targets); SGTF has served as a proxy in the United Kingdom for identifying B.1.1.7 cases (*1*).

**TABLE:** Characteristics of SARS-CoV-2 variants of concern — worldwide, September 2020–January 2021

Variant designation	First identification		Characteristic mutations (protein: mutation)	No. of current sequence-confirmed cases		No. of countries with sequences
	Location	Date		United States	Worldwide	
B.1.1.7 (20I/501Y.V1)	United Kingdom	Sep 2020	ORF1ab: T1001I, A1708D, I2230T, del3675–3677 SGF	76	15,369	36
			S: del69–70 HV, del144 Y, N501Y, A570D, D614G, P681H, T761I, S982A, D1118H			
			ORF8: Q27stop, R52I, Y73C			
			N: D3L, S235F			
B.1.351 (20H/501Y.V2)	South Africa	Oct 2020	ORF1ab: K1655N	0	415	13
			E: P71L			
			N: T205I			
			S:K417N, E484K, N501Y, D614G, A701V			
P.1 (20J/501Y.V3)	Brazil and Japan	Jan 2021	ORF1ab: F681L, I760T, S1188L, K1795Q, del3675–3677 SGF, E5662D	0	35	2
			S: L18F, T20N, P26S, D138Y, R190S, K417T, E484K, N501Y, D614G, H655Y, T1027I			
			ORF3a: C174G			
			ORF8: E92K			
			ORF9: Q77E			
			ORF14: V49L			
			N: P80R			

**Abbreviations:** del = deletion; E = envelope protein; N = nucleocapsid protein; ORF = open reading frame; S = spike protein.

Multiple lines of evidence indicate that B.1.1.7 is more efficiently transmitted compared with other SARS-CoV-2 variants circulating in the United Kingdom. U.K. regions with a higher proportion of B.1.1.7 sequences had faster epidemic growth than did other areas, diagnoses with SGTF increased faster than did non-SGTF diagnoses in the same areas, and a higher proportion of contacts were infected by index patients with B.1.1.7 infections than by index patients infected with other variants (*1*,*3*).

Variant B.1.1.7 has the potential to increase the U.S. pandemic trajectory in the coming months. To illustrate this effect, a simple, two-variant compartmental model was developed. The current U.S. prevalence of B.1.1.7 among all circulating viruses is unknown but is thought to be <0.5% based on the limited number of cases detected and SGTF data (*8*). For the model, initial assumptions included a B.1.1.7 prevalence of 0.5% among all infections, SARS-CoV-2 immunity from previous infection of 10%–30%, a time-varying reproductive number (R_t_) of 1.1 (mitigated but increasing transmission) or 0.9 (decreasing transmission) for current variants, and a reported incidence of 60 cases per 100,000 persons per day on January 1, 2021. These assumptions do not precisely represent any single U.S. location, but rather, indicate a generalization of conditions common across the country. The change in R_t_ over time resulting from acquired immunity and increasing prevalence of B.1.1.7, was modeled, with the B.1.1.7 R_t_ assumed to be a constant 1.5 times the R_t_ of current variants, based on initial estimates from the United Kingdom (*1*,*3*). 

Next, the potential impact of vaccination was modeled assuming that 1 million vaccine doses were administered per day beginning January 1, 2021, and that 95% immunity was achieved 14 days after receipt of 2 doses. Specifically, immunity against infection with either current variants or the B.1.1.7 variant was assumed, although the effectiveness and duration of protection against infection remains uncertain, because these were not the primary endpoint of clinical trials for initial vaccines.

**FIGURE 1:**
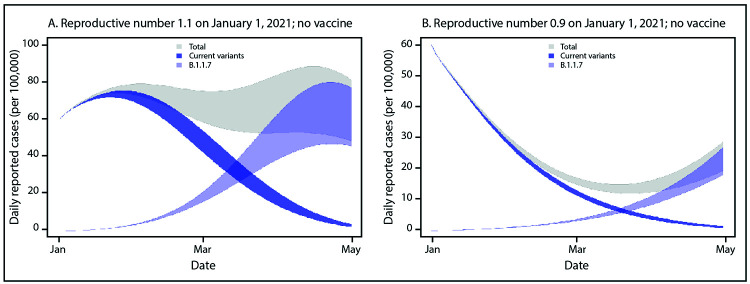
Simulated case incidence trajectories* of current SARS-CoV-2 variants and the B.1.1.7 variant,^†^ assuming no community vaccination and either initial Rt = 1.1 (A) or initial Rt = 0.9 (B) for current variants — United States, January–April 2021 **Abbreviation:** R_t_ = time-varying reproductive number. * For all simulations, it was assumed that the reporting rate was 25% and that persons who were seropositive or infected within the simulation became immune. The simulation was initialized with 60 reported cases of SARS-CoV-2 infection per 100,000 persons (approximately 200,000 cases per day in the U.S. population) on January 1, 2021. Bands represent simulations with 10%–30% population-level immunity as of January 1, 2021. ^†^ Initial B.1.1.7 prevalence is assumed to be 0.5% among all infections and B.1.1.7 is assumed to be 50% more transmissible than current variants.

**FIGURE 2:**
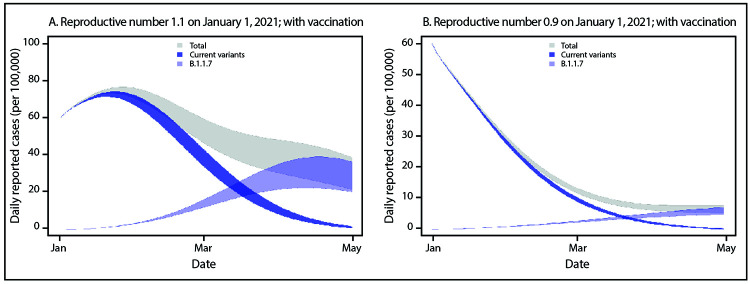
Simulated case incidence trajectories* of current SARS-CoV-2 variants and the B.1.1.7 variant,^†^ assuming community vaccination^§^ and initial R_t_ = 1.1 (A) or initial R_t_ = 0.9 (B) for current variants — United States, January–April 2021 **Abbreviation:** R_t_ = time-varying reproductive number. * For all simulations, it was assumed that the reporting rate was 25% and that persons who were seropositive or infected within the simulation became immune. The simulation was initialized with 60 reported cases of SARS-CoV-2 infection per 100,000 persons (approximately 200,000 cases per day in the U.S. population) on January 1, 2021. Bands represent simulations with 10%–30% population-level immunity as of January 1, 2021. ^†^ Initial B.1.1.7 prevalence is assumed to be 0.5% among all infections and B.1.1.7 is assumed to be 50% more transmissible than current variants. ^§^ For vaccination, it was assumed that 300 doses were administered per 100,000 persons per day (approximately 1 million doses per day in the U.S. population) beginning January 1, 2021, that 2 doses achieved 95% immunity against infection, and that there was a 14-day delay between vaccination and protection.

In this model, B.1.1.7 prevalence is initially low, yet because it is more transmissible than are current variants, it exhibits rapid growth in early 2021, becoming the predominant variant in March (Figure 1). Whether transmission of current variants is increasing (initial R_t_ = 1.1) or slowly decreasing (initial R_t_ = 0.9) in January, B.1.1.7 drives a substantial change in the transmission trajectory and a new phase of exponential growth. With vaccination that protects against infection, the early epidemic trajectories do not change and B.1.1.7 spread still occurs (Figure 2). However, after B.1.1.7 becomes the dominant variant, its transmission was substantially reduced. The effect of vaccination on reducing transmission in the near term was greatest in the scenario in which transmission was already decreasing (initial R_t_ = 0.9) (Figure 2). Early efforts that can limit the spread of the B.1.1.7 variant, such as universal and increased compliance with public health mitigation strategies, will allow more time for ongoing vaccination to achieve higher population-level immunity.

## Discussion

Currently, there is no known difference in clinical outcomes associated with the described SARS-CoV-2 variants; however, a higher rate of transmission will lead to more cases, increasing the number of persons overall who need clinical care, exacerbating the burden on an already strained health care system, and resulting in more deaths. Continued genomic surveillance to identify B.1.1.7 cases, as well as the emergence of other variants of concern in the United States, is important for the COVID-19 public health response. Whereas the SGTF results can help identify potential B.1.1.7 cases that can be confirmed by sequencing, identifying priority variants that do not exhibit SGTF relies exclusively on sequence-based surveillance.

The experience in the United Kingdom and the B.1.1.7 models presented in this report illustrate the impact a more contagious variant can have on the number of cases in a population. The increased transmissibility of this variant requires an even more rigorous combined implementation of vaccination and mitigation measures (e.g., distancing, masking, and hand hygiene) to control the spread of SARS-CoV-2. These measures will be more effective if they are instituted sooner rather than later to slow the initial spread of the B.1.1.7 variant. Efforts to prepare the health care system for further surges in cases are warranted. Increased transmissibility also means that higher than anticipated vaccination coverage must be attained to achieve the same level of disease control to protect the public compared with less transmissible variants.

In collaboration with academic, industry, state, territorial, tribal, and local partners, CDC and other federal agencies are coordinating and enhancing genomic surveillance and virus characterization efforts across the United States. CDC coordinates U.S. sequencing efforts through the SARS-CoV-2 Sequencing for Public Health Emergency Response, Epidemiology, and Surveillance (SPHERES)^§§^ consortium, which includes approximately 170 participating institutions and promotes open data-sharing to facilitate the use of SARS-CoV-2 sequence data. To track SARS-CoV-2 viral evolution, CDC is implementing multifaceted genomic surveillance to understand the epidemiologic, immunologic, and evolutionary processes that shape viral phylogenies (phylodynamics); guide outbreak investigations; and facilitate the detection and characterization of possible reinfections, vaccine breakthrough cases, and emerging viral variants. In November 2020, CDC established the National SARS-CoV-2 Strain Surveillance (NS3) program to improve the representativeness of domestic SARS-CoV-2 sequences. The program collaborates with 64 U.S. public health laboratories to support a genomic surveillance system; NS3 is also building a collection of SARS-CoV-2 specimens and sequences to support public health response and scientific research to evaluate the impact of concerning mutations on existing recommended medical countermeasures. CDC has also contracted with several large commercial clinical laboratories to rapidly sequence tens of thousands of SARS-CoV-2–positive specimens each month and has funded seven academic institutions to conduct genomic surveillance in partnership with public health agencies, thereby adding substantially to the availability of timely genomic surveillance data from across the United States. In addition to these national initiatives, many state and local public health agencies are sequencing SARS-CoV-2 to better understand local epidemiology and support public health response to the pandemic.

The findings in this report are subject to at least three limitations. First, the magnitude of the increase in transmissibility in the United States compared with that observed in the United Kingdom remains unclear. Second, the prevalence of B.1.1.7 in the United States is also unknown at this time, but detection of variants and estimation of prevalence will improve with enhanced U.S. surveillance efforts. Finally, local mitigation measures are also highly variable, leading to variation in R_t_. The specific outcomes presented here are based on simulations and assumed no change in mitigations beyond January 1. 

The increased transmissibility of the B.1.1.7 variant warrants rigorous implementation of public health strategies to reduce transmission and lessen the potential impact of B.1.1.7, buying critical time to increase vaccination coverage. CDC’s modeling data show that universal use of and increased compliance with mitigation measures and vaccination are crucial to reduce the number of new cases and deaths substantially in the coming months. Further, strategic testing of persons without symptoms of COVID-19, but who are at increased risk for infection with SARS-CoV-2, provides another opportunity to limit ongoing spread. Collectively, enhanced genomic surveillance combined with increased compliance with public health mitigation strategies, including vaccination, physical distancing, use of masks, hand hygiene, and isolation and quarantine, will be essential to limiting the spread of SARS-CoV-2 and protecting public health.

**Summary**

**What is already known about this topic?**

A more highly transmissible variant of SARS-CoV-2, B.1.1.7, has been detected in 12 U.S. states.

**What is added by this report?**

Modeling data indicate that B.1.1.7 has the potential to increase the U.S. pandemic trajectory in the coming months. CDC’s system for genomic surveillance and the effort to expand sequencing will increase the availability of timely U.S. genomic surveillance data.

**What are the implications for public health practice?**

The increased transmissibility of the B.1.1.7 variant warrants universal and increased compliance with mitigation strategies, including distancing and masking. Higher vaccination coverage might need to be achieved to protect the public. Genomic sequence analysis through the National SARS-CoV-2 Strain Surveillance program will enable a targeted approach to identifying variants of concern in the United States.

## Footnotes

* https://www.gov.uk/government/news/phe-investigating-a-novel-variant-of-covid-19.

† https://www.cdc.gov/coronavirus/2019-ncov/transmission/variant-cases.html.

§ https://www.japantimes.co.jp/news/2021/01/11/national/science-health/new-coronavirus-variant-japan/.

¶ https://www.fda.gov/news-events/press-announcements/fda-issues-alert-regarding-sars-cov-2-viral-mutation-health-care-providers-and-clinical-laboratory?utm_medium.

** https://virological.org/t/spike-e484k-mutation-in-the-first-sars-cov-2-reinfection-case-confirmed-in-brazil-2020/584.

†† https://www.cdc.gov/coronavirus/2019-ncov/more/science-and-research/scientific-brief-emerging-variants.html.

§§ https://www.cdc.gov/coronavirus/2019-ncov/covid-data/spheres.html.
